# Pathways and crossroads to creditions: Insights from a retrospective view

**DOI:** 10.3389/fpsyg.2022.942590

**Published:** 2022-11-04

**Authors:** Hans-Ferdinand Angel

**Affiliations:** Karl Franzens University of Graz, Graz, Austria

**Keywords:** credition, research development, basic research, model of credition, religiosity

Credition is a neologism derived from the Latin word credere (to believe) and designates processes of believing (Angel, 2013a). In many languages (and esp. in German) the term belief is widely associated with religion and religious beliefs. Indeed, the need for a new term became evident during the so-called Regensburg Symposia (1998–2005) (Angel, 2006a) that were aimed at increasing our understanding of the phenomenon of religiosity (see below) and the dynamics of ‘religious beliefs’. Given this background, it is important to emphasize that credition is neither a religious nor a theological term. Rather, it was coined as a psychological term in analogy to other psychological terms including cognition, emotion, and volition. No religion is needed in order to understand “credition,” but knowledge about credition may help us to better understand religious beliefs. Although the intention of this article is to point to issues which appeared as crossroads and pathways in the emerging history of creditions, it does not present a chronology of events but focusses on theoretical issues.

## Precursory hermeneutics as crossroads to credition

### Blind spot time-related beliefs

Talking about belief or credition means initially to talk about notions, i.e., the notion of belief and the notion of credition. Both terms highlight related but different phenomena. Belief has been a topic discussed since Antiquity. It might have contributed to our lack of understanding that something like fluid or temporally evolving believing processes might exist because, at least since late Antiquity and the early Middle Ages, the predominant scientific practice has been to talk about ‘belief’ as a static entity, i.e., as a noun (Angel, 2022a). But on a linguistic level we must address the relation between a noun and its corresponding verb. To proceed from a noun related understanding of beliefs to an action-based understanding of believing processes that can be expressed in terms of a verb (e.g., while believing) requires a paradigm shift. The paradigm shift that underpins going from understanding beliefs to understanding the processes of believing is a precondition for the concept of credition that must be elaborated within the intersection of different scholarly fields such as linguistics, epistemology, philosophy of mind, cognitive science, neuroscience, sociology, information theory, psychology, and psychology of religion (Angel et al., 2017).

### Language related issues

For understanding beliefs and following the path of the paradigm shift, a crucial issue turned out to be the language in which the shift was discussed. Certain terms are central to the credition concept: meaning, mind, perception, evidence, and representation. These terms could be obstacles to understanding believing processes. This shall be exemplified by two lexemes.

(a) Ancient Greek offers two words to express the notion of the verb ‘to believe’: δoξάζει*ν* [doxázein] and π*ιστε*úε*ιν* [pistéuein]. In both cases the relation between noun and verb can be discussed because both verbs have corresponding nouns: δóξα [dóxa] and $\pi \overset{´}{\iota }\sigma \tau \iota \varsigma$ [pístis]. The former has been used since the 4th century to express the correct ‘orthodox’ Christian faith ['oρθóς: orthós: correct]. However, the relation between the noun and verb is awkward in Latin. The Latin translation for ‘pístis’ is ‘fides’, from which stems the English term ‘faith’. The required paradigm shift is impeded because there are no corresponding verbs for the Latin “fides” or the English “faith.” To switch from a substantive expression (fides; faith) to a verbal expression, one must change the wordstem (credere, to believe). Another obstacle is that for the English verb “to believe” the noun “belief” exists, whereas the Latin term “credere” lacks a correspondent noun. This lack contributed to the blind spot because until the Renaissance Western philosophy was based on the use of Latin. Also, the English language has two nouns – “belief” and “faith.” In contrast, the German language provides only one noun (“Glaube”) which covers the semantic broadness of both English nouns. Because there is no verb for the English “faith,” often the adjective ‘religious’ is used to express “having faith.”

(b) Specific links between “religion,” “faith,” and “belief” are apparent. The semantic broadness of ‘religion’ leads to an impervious terminological mess which deeply infects the research on credition. Research on religions and their role in societies began to flourish in the late 1800's in different ways. After Darwin (1859) an interest in the evolution of religion was fostered (Feierman and Oviedo, 2019); the psychology of religion beginnings included neuropsychological perspectives (James, 1902); and the sociology of religion started to examine the social role of religions (Durkheim, 1912). The 19th-century-debates spread the term religion widely, contributing to its present dominant appeal (Seitz and Angel, 2014). This is apparent in the names of certain scholarly sub-disciplines such as history of religion, psychology of religion, sociology of religion, philosophy of religion, and phenomenology of religion. This predominance of ‘religion’ causes at least three problems including (1) a marginalization of the term religiosity, (2) the absence of an academic goal to clarify the terms religiosity or religiousness (Angel, 2013b), and (3) the absence of ‘religiosity’ as theoretical starting point so that many important issues in understanding religious behavior – be it dysfunctional or not – cannot be addressed in a theoretically adequate manner (Seitz et al., 2021). But any theoretically sound understanding of ‘religious experience’ has to encompass three elements – religion, religiosity, and the individual or collective relation between religion and religiosity (Angel, 2019). Importantly, the nucleus of all later development of the idea of credition is routed in the German language. The Regensburg Symposia helped us to better understand the German term “Religiosität,” not “Religion” (Angel, 2006a). “Religiosität” as typical German term cannot be adequately translated into English because there exist three terms – religiosity, religiousness, and spirituality – none of which is fully equivalent to the German term Religiosität. The book-title ‘Geschichte der Religiosität im Mittelalter’ (Angenendt, 2009) cannot be translated into English in a satisfying manner.

### Semantic of (religious) belief(s)

A second – rarely addressed (Sharpe, 1983, p. VIII)“–'problem is the neglect of the linguistic nature of “religious” as an adjective. ‘Religious’ as an adjective has dual associations: it can be related to two nouns – “religion” and “religiosity.” Because associating religious with religion is widely accepted in empirical scientific research, the dual character of the adjective is less apparent. A striking example is the adjective interreligious. Because it is typically associated with different religions, its other function is often lost so that it is seldom invoked to appreciate the different features of religiosity or religiousness as might be possible when considering, for example, open-mindedness vs. fundamentalism. “The common language use seems to put the terms religious, religiosity, and religion in a melting pot from where the words can be taken out in an exchangeable manner” (Seitz et al., 2021, p. 62).

In recent years linguistic philosophy has pointed to the important role of languages in the production of worldviews (Rorty, 1967; Waismann, 1997). The role of languages is also of crucial importance for the interdisciplinary and global credition research project and was prominent in the attempts to conceptualize credition. Thus, it is not mere storytelling when the complicated linguistic issues in the topic of beliefs in general and specifically religious beliefs are highlighted. Three types of issues emerged in relation to the languages used.

(a) It is possible to clarify the relation between nouns and verbs in Indo-European languages, but not in all languages. There are restrictions in generative grammar and its later developments (Chomsky, 1965, 1986). More advanced ontogenetically based linguistic theories (Tomasello, 2003, 2008) must be integrated into credition research (Seitz et al., 2018), and the role of participles influences the linguistic possibilities. In an Anglo-American but not in a German context, ‘believing’ can be used in the same manner as is ‘learning’, prompting discussion of how the cognitive processes of believing and learning are related.

(b) The chaotic religious semantics reflects the ambiguity of its emotional loading. At least in the context of Western thinking it might be adequate to conceive of religion as “an incredibly powerful catalyst for both our best and worst” (Sapolsky, 2017, p. 621). This ambiguity exposes the emotional loading of ‘religious’ beliefs, and thus the topic of belief in general.

(c) The English ‘belief’ can be used in a plural form (beliefs), whereas no plural exists for the German “Glaube.” This distinction is often explicitly highlighted when epistemic texts are to be translated. For the translation of ‘belief’ into German, sometimes the term “Meinung” is used instead of “Glaube” (Bieri, 1987, p. 106). But since “Meinung” also conveys the English equivalent “opinion,” an identity of belief and opinion is implied which makes it difficult to convey the role of trust in believing. Such a translation follows the Latin speaking trend in philosophy, as the Latin term “credere” does not include the notion of trust, whereas the Greek term “pisteuein” does. This trend, rooted in Latin, tends to end up in the field of epistemology.

(d) Scientific research is often driven by a WEIRD (Western, educated, industrialized, rich, democratic) perspective (Henrich et al., 2010). When developing credition in a globalized context this restricted perspective must be overcome. We need to develop sensitivity to the richness and different mental and emotional roots of non-European languages, as van Leeuwen demonstrates when he compares Fante, Thai, and Mandarin (van Leeuwen et al., 2022).

### Milestones of hermeneutic clarification

The research on credition happens in the collaboration of different disciplines with different methodologies and language rules (Wittgenstein, 1953). A considerable amount of preliminary hermeneutic clarifications is needed to comprehend the theoretical groundwork that underpins the credition concept and its neurophysiological base. Learning about credition might appear to be a challenge (Madzarevic, 2022).

(a) “Conceptional questions antecede matters of truth and falsehood. […] Hence conceptual questions are not amendable to scientific investigation and experimentation. […] Distinguishing conceptual questions from empirical ones is of first importance” (Bennett and Hacker, 2007, p. 2). Many terms which are embedded in concepts are relevant for an understanding of credition, such as process, function, action, relation (Seitz et al., 2018, p. 1257f.), normal and normality, meaning, value, will and free will, decision and decision-making, and others.

(b) The most adequate synonym for credition seems to be process of believing. Nevertheless, this is correct only in comparison to a static and noun-related understanding. Process is the antonym to state. But in the context of cognitive neuroscience, credition processes must be differentiated from the functions of credition that are attributed to these processes. Here process is the antonym to function.

(c) The term credition can be found in both singular and plural forms in the literature. In its singular use it designates a generic term in analogy to cognition, emotion, volition, and similar terms. When it is used in plural, the intention is to point to the neurophysiological processes that are occurring while someone is believing (Angel, 2022a).

(d) The relation between belief and process of believing can be expressed in mathematical terms: B = f(b,t). This means that belief (B) is a function of believing (b) and the character of a ‘belief’ depends on what has occurred across the time (t) (Seitz et al., 2018, p. 1257).

## Crossroads to an understanding of creditions as brain function

### Blind spot neural believing processes

Credition as an idea emerged during the Regensburg Symposia. It was inspired by ongoing debates about the origin of religious beliefs. In cognitive neuroscience two seemingly incompatible and camp-building positions which seemed to be based on two different psychological concepts were held. The limbic marker theory suggests “that the primary substrate for this <religious and mystical; HFA> experience is the limbic system” and “predicts that functional neuroimaging during numinous experiences in individuals who have repeated religious transports would reveal alterations in limbic system activity” (Saver and Rabin, 1999, p. 204). In contrast, a cortical marker theory suggests “that religious experience may be a cognitive process, mediated by a pre-established neural circuit, involving dorsolateral prefrontal, dorsomedial frontal and medial parietal cortex” (Azari et al., 2001, p. 1651). For understanding “Religiosität” cognition and emotion appeared as insufficient categories and believing processes (i.e., creditions) were postulated (Angel, 2006b, p. 71).

The idea of credition was then not more than a postulate, but it allowed us to address believing processes by means of neuroscientific approaches (Seitz, 2017, p. 2022). The paradigm-shift toward understanding beliefs as manifestations of processes faced a similar situation because beliefs had typically been analyzed hermeneutically. A PubMed-review of empirical findings revealed “a lack of empirical effort to understand belief” (Seitz and Angel, 2012) which is sometimes addressed even as “neglect of belief” (Connors and Halligan, 2015).

### Milestones toward credition

Beliefs differ from knowledge because they imply subjective meaning. Thus, one key issue for understanding believing is centered on the role of emotional valuations and subjective meaning-making. A series of publications emphasizes relevant aspects of this (Angel, 2022b).

**Figure 1:**
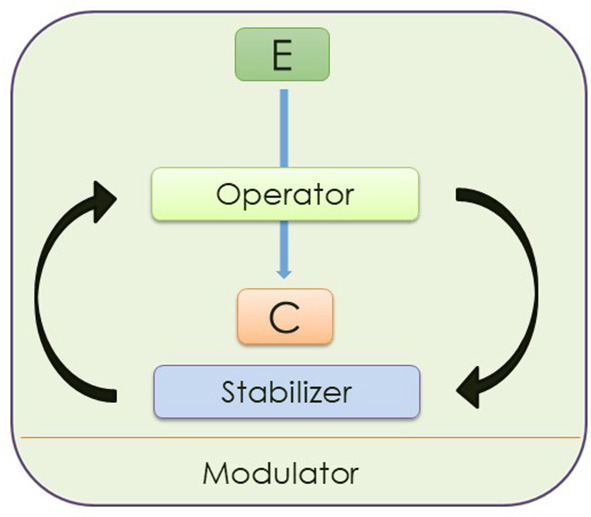
The credition model describing the process of believing. The “Enclosure Function” (E) defines the enclosed representation of the perceived stimulus, the “Converter Function”(C) provides the appropriate action in response to the stimulus. By reinforcement learning the putative beliefs are stabilized which is indicated by the “Stabilizer Function.” These three supramodal functions are modulated by the internal state of the individual–called “Modulator Function.” In the figure the different type of the modulator function is indicated by a thin line. © HF Angel; conference presentation 2012, for the first time published in SFU Research Bulletin, 3/1, 1–20 Angel and Seitz (2016).

(a) Like other cognitive processes, the process character of credition includes several different mental operations that are heavily involved in the perception of events or objects in the outer world and in control of behavior (Angel and Seitz, 2016). As shown in Figure 1, this multifunctionality can be specified (Angel, 2017). The so-called enclosure function denotes the self-organizing probabilistic assembly of mental attributes of a given object or event that a person is encountering into a coherent mental construct (Angel and Seitz, 2016). Beliefs can lead to action (converter function) and are stabilized by reinforcement learning (stabilizer function). These supramodal functions are modified by the individuality of agents (modulator function). The “functional anatomy” of the believing process can be described at a neurophysiological level (Seitz, 2017).

(b) Believing can be explained by a dual-component model which combines self-organization process of cognitive and emotional elements with a belief evaluation component. Subjective representations encompass self-cognition that refers to a multi-layered self on a physical, interpersonal, and higher social level. A major role plays the right dorsolateral prefrontal cortex (DLPFC) and the medial frontal cortex (MFC) (Sugiura et al., 2015).

(c) To connect the neuroscientific aspect with general anthropological dimensions of believing, the role of emotions in meaning-making was included (Paloutzian and Mukai, 2017). It is suggested that the formation of belief systems and their behavioral consequences can be predicted as result of a probabilistic perception-action-valuation model which represents the mental operations that seem to underly believing processes (Seitz et al., 2016).

(d) Beliefs are “the neuropsychic product of fundamental brain processes that attribute affective meaning to concrete objects and events, enabling individual goal setting, decision making and maneuvering in the environment” which can be categorized as empirical, relational, and conceptual beliefs. “Whilst empirical beliefs about objects and relational beliefs about events develop below the level of awareness and are up-dated dynamically conceptual beliefs are more complex as being based on narratives and participation in ritual acts” (Seitz and Angel, 2020, p. 1).

(e) This allows us to hypothesize that the ‘capacity of believing’ is a result of the evolution of the brain. The parietal cortex which accommodates in close vicinity the neural representations of executive, perceptual, and higher order conceptual functions may be a candidate area (Seitz, 2022).

(f) Beliefs are constantly adjusted by the perception of new signals in a Bayesian sense and can be explained as result of believing processes which include learning. They take place on a neurological level but integrate information which have been perceiving from the social environment. Note, the general model results in a mathematically expressed equation:


$$\begin{array}{l} \text{B}=\text{S}/\text{N}\times \text{V}+\left(\alpha \times \delta \right)\times {\text{V}}_{\text{δ}}.\;\left[\text{Seitzetal }2018,1259\right]. \end{array}$$

(g) From a clinical perspective believing processes can become dysfunctional (Seitz, 2021). This may play a role in psychiatric contexts (Paloutzian et al., 2018) and have an impact on religious beliefs (Seitz et al., 2021).

(h) Maintaining beliefs is interwoven with memory functions in a multifaceted fashion. For instance, linking the typically rapid and adequate reactions of a person to what he or she believes is enabled by working memory. Perceptions are stored in episodic memory as beneficial or aversive events, while the corresponding verbal descriptions of what somebody believes are stored in semantic memory. After recall from memory of what someone believes, personally relevant information can be communicated to other people (Seitz et al., 2022a).

(i) The Credition project follows three research strands: basic (2011), applied (2014), and implementation (2016). The CreditionLab (opened 2018 at the University of Technology in Graz) tests the so called ‘model of credition’ as specific communication tool intended to make visible the functionality of believing (Angel and Seitz, 2016; Angel, 2017) and seems successfully applied as reference tool for communication-settings (Mitropoulou, 2017; Mitropoulou et al., 2018; Hick et al., 2020; Kranabitl et al., 2021; Lumbreras et al., 2021; Tietz et al., 2022).

## Discussion

**Figure 2:**
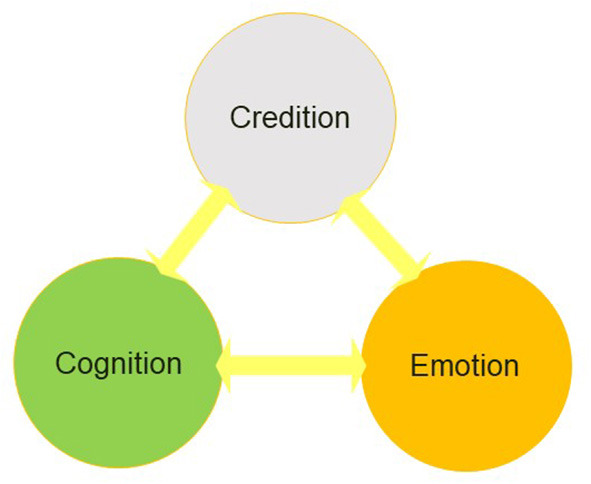
The figure shows the interdependence of credition, emotion, and cognition as originally depicted in the basic model of credition. [© HF Angel].

During the Regensburg Symposia it became necessary to establish credition linguistically as scientific term (Angel, 2006a), although from the beginning creditions were phenomenologically conceived as intertwined with cognition and emotion (Angel, 2016, 2021) (Figure 2: basic model).

Because credition is now a widely used term, the relations between creditions and other emotional and cognitive processes can be addressed (Seitz et al., 2018). As believing is intimately linked with inferential information processing, information that is processed and/or modified in the brain will be labeled with diverse attributions. Typically, these attributions correspond to meta-cognitive self-attributions or third-persons attributions concerning behavior observed in other people (Seitz et al., 2022b). Such (*post-hoc*) attributions are conceptually different from the belief categories that have been defined with respect to the type of information processed (Seitz and Angel, 2020).

To construct the “model of credition,” conceptional neurophysiological findings about believing were translated into model-specific terms. Their adequateness and the methodical transformation of the underlying concepts may be discussed. For instance, the production process was inspired by neurophysiological findings about the simultaneous production of cognitive and emotional processes in the prefrontal-medial cortex (Gray et al., 2002; Schaefer and Gray, 2007). Since no term existed to express linguistically this simultaneity “bab” as basic term of the model of credition was coined. It designates: “emotional loaded proposition.”

For stable beliefs it might be adequate to talk about religious, political, or economic beliefs. From a processual perspective, that is from a credition perspective, such characteristics do not make sense (Angel 2022a, 615–621).

## Author contributions

The author confirms being the sole contributor of this work and has approved it for publication.

## Funding

This paper was funded by Dr. Rüdiger Seitz, *via* the Volkswagen Foundation, Siemens Healthineers, and the Betz Foundation.

## Conflict of interest

The author declares that the research was conducted in the absence of any commercial or financial relationships that could be construed as a potential conflict of interest.

## Publisher's note

All claims expressed in this article are solely those of the authors and do not necessarily represent those of their affiliated organizations, or those of the publisher, the editors and the reviewers. Any product that may be evaluated in this article, or claim that may be made by its manufacturer, is not guaranteed or endorsed by the publisher.
